# The Relationship Between Personality Traits and Entrepreneurial Intention Among College Students: The Mediating Role of Creativity

**DOI:** 10.3389/fpsyg.2022.822206

**Published:** 2022-02-03

**Authors:** Li-Na Li, Jian-Hao Huang, Sun-Yu Gao

**Affiliations:** Dhurakij Pundit University, Bangkok, Thailand

**Keywords:** entrepreneurial intention, personality traits, creativity, college students, mediation

## Abstract

Significant research has been conducted on the influence of entrepreneurial intention on entrepreneurial education and entrepreneurship practice. Similarly, this study aims to explore how creativity plays a mediating role in the influence of personality traits on entrepreneurial intention. As many as 674 valid questionnaires were collected from college students in China, allowing the relationship between personality traits, creativity, and entrepreneurial intention to be analyzed in detail. The following results are found through a series of explorations. First, neuroticism in personality traits has a significant negative impact on entrepreneurial intention, while conscientiousness, openness, and extraversion have a significant positive impact. Second, neuroticism has a significant negative impact on creativity, while conscientiousness, openness, and extraversion have a significant positive impact. Third, creativity has a significant positive impact on entrepreneurial intention, it has a partial mediating role between neuroticism, conscientiousness, extraversion, and entrepreneurial intention along with a complete mediating role between openness and entrepreneurial intention. The research results further provide a reference value for the improvement and optimization of entrepreneurial practice.

## Introduction

Entrepreneurship has a significant impact on a country or region’s economic, political, and social environment (Montiel and Clark, 2018). Entrepreneurship is a process that goes from idea to practice and from intention to implementation. A significantly positive correlation exists between entrepreneurial intention and entrepreneurial behavior (Kong et al., 2020). College students are often considered to be the potential targets of entrepreneurship. It is consequently necessary to improve their entrepreneurial intention (Veciana et al., 2005). The term entrepreneurial intention refers to the belief that an individual plans to start a new company in the future (Thompson, 2009), which exerts a significant predictive effect on entrepreneurial behavior (Boubker et al., 2021; Elnadi and Gheith, 2021).

Among the factors that influence entrepreneurial intention, personality traits are a significant one (Şahin et al., 2019; Ndovela and Chinyamurindi, 2021). Personality traits refer to the unchanging and stable psychological traits that individuals possess (Costa and McCrae, 1992). According to the existing research, the Big-Five Personality is one of the most comprehensive personality classifications available (Singh and DeNoble, 2003). Relevant empirical research has also found that personality traits have a significant impact on entrepreneurial intention (Şahin et al., 2019; Wu and Wu, 2020; Awwad and Al-Aseer, 2021; Khan et al., 2021). Research on the influence of personality traits on entrepreneurial intention has a great impact on improving university students’ entrepreneurial intention.

Previous studies on entrepreneurial intentions have reported a mediating role of creativity (Imran et al., 2018; Danish et al., 2019; Chaubey et al., 2021). Murad et al. (2021) found that creativity significantly and positively impacts entrepreneurial intentions. Hu et al. (2018) found that entrepreneurial alertness exerts a mediating effect on the relationship among creativity, proactive personality, and entrepreneurial intention. Hamidi et al. (2008) suggested that creativity should be included as a predictor variable in empirical research on entrepreneurial intentions. Different personality traits may have different effects on creativity. For example, neuroticism has a significant negative impact on creativity. Influential people with high openness are more creative but exhibit less agreeableness (Karwowski and Lebuda, 2016). Moreover, conscientiousness, extroversion, and agreeableness positively and significantly impact creativity (Mumford, 2011). Openness has a positive and significant impact on creativity (Grohman et al., 2017; Jirásek and Sudzina, 2020; Theurer et al., 2020). Creative people can maintain a positive attitude and a high degree of self-confidence when starting a business (Zhao et al., 2005). Some empirical studies have shown that creativity positively and significantly impacts entrepreneurial intentions (Shi et al., 2020; Murad et al., 2021; Tantawy et al., 2021). Personality traits may influence entrepreneurial intentions through creativity. Considering research gaps and practical needs, this research intends to explore the influence of personality traits on entrepreneurial intentions and the mediating role of creativity between personality traits and entrepreneurial preferences.

In summary, the present study has two objectives. First, considering that most of the previous research samples were from Western countries (Şahin et al., 2019; Awwad and Al-Aseer, 2021; Khan et al., 2021) and the vast differences between Chinese and Western cultures, the current study utilizes Chinese samples for verification. Second, this research explores the influence path of personality traits on entrepreneurial intentions from the perspective of mediation. Therefore, this research uses personality traits as the independent variable, creativity as the mediating variable, and entrepreneurial intention as the dependent variable to explore the influence mechanism between personality traits, creativity, and entrepreneurial intentions. The study not only provides suggestions for improving college students’ entrepreneurial intention but also summarizes the limitations and future directions, providing a solid foundation for future research.

## Literature Review and Hypothesis Development

### A Cross-Cultural Cognitive Model of New Venture Creation

This research uses a cross-cultural cognitive model of new venture creation as the theoretical basis to understand the influence of personality traits, creativity, and entrepreneurial intentions. The model highlights that the social background, cultural value, and personal factors can affect cognition, cognition can affect entrepreneurial intention, and cognition can affect entrepreneurial intentions through personal factors (Busenitz and Lau, 1996). The personality traits in this study can be regarded as personal factors. Studies have indicated that cognition is closely related to creativity (Sternberg and Lubart, 1999). Studies have also pointed out that cognition is the process of creativity formation (Runco and Chand, 1995; James et al., 2010). Therefore, creativity can be regarded as cognition. Empirical studies have shown that the personality traits significantly affect cognition (Schaie et al., 2004), and cognition has a positive and significant impact on entrepreneurial intentions (Bian et al., 2021). A**n** empirical study by Liu and Wei (2003) found that the personal factors can significantly affect cognition, and cognition can significantly affect entrepreneurial **organization** and expansion intentions. Therefore, this study concludes that the personality traits can significantly affect creativity, thus significantly affecting entrepreneurial intentions. In other words, creativity may have an intermediary effect between personality traits and entrepreneurial intentions. The results of this research can extend the application of cross-cultural cognitive models of startups and enhance the richness of their theoretical models and provide empirical research support for this theoretical model.

### Personality Traits and Entrepreneurial Intention

Previous studies have found that the personality traits are closely related to entrepreneurial intentions (Şahin et al., 2019; Arru, 2020; Bazkiaei et al., 2020). Personality traits are suitable for evaluating suitability for entrepreneurship (Zhao et al., 2010). Wang et al. (2016) proposed that the personality traits significantly impact individuals aiming to become entrepreneurs. Moreover, personality traits significantly impact the cognitive model of entrepreneurial intention (Karabulut, 2016; Fragoso et al., 2020). Different personality traits have different effects on entrepreneurial intentions. For example, neuroticism has a significant negative impact on entrepreneurial intentions, conscientiousness, extraversion, and agreeableness, whereas openness has positive and significant effects on entrepreneurial intentions (Israr and Saleem, 2018; Khan et al., 2021). High extroversion, openness, and conscientiousness have a strong impact on entrepreneurial intentions, whereas neuroticism and agreeableness have a weak impact on entrepreneurial intentions (Zhao et al., 2010; Brandstatter, 2011; Liang et al., 2015). Therefore, this research proposes the following hypothesis:

H1: The personality traits of college students significantly impact entrepreneurial intentions.

### Personality Traits and Creativity

Many empirical studies have found that the personality traits are closely related to creativity (Hamedinasab and Azizi, 2021; Sarma and Borooah, 2021). Among these traits, neuroticism has a significant negative impact on creativity (Amin et al., 2020; Shokrkon and Nicoladis, 2021), whereas conscientiousness, extraversion, and agreeableness positively and significantly impact creativity (Mumford, 2011). Openness has a positive and significant impact on creativity (Jirásek and Sudzina, 2020; Theurer et al., 2020). Extraversion has a positive and significant impact on creativity (Russ, 2003). In addition, a study by Amin et al. (2020) found that neuroticism has a negative and significant impact on creativity, whereas openness, extroversion, and agreeableness have a positive and significant impact on creativity. People with high openness are more creative but exhibit less agreeableness (Feist, 1999; Silvia et al., 2012; Karwowski et al., 2013; Karwowski and Lebuda, 2016). In conclusion, personality traits are an essential factor for creativity (Woodman et al., 1993). Therefore, this research proposes the following hypothesis:

H2: The personality traits of college students have a significant impact on creativity.

### Creativity and Entrepreneurial Intention

Creativity has been found to be highly correlated with entrepreneurial intentions (Bellò et al., 2018; Entrialgo and Iglesias, 2020). Moreover, some researchers have pointed out that creativity should be considered an essential resource for entrepreneurs (Ahlin et al., 2014; Khedhaouria et al., 2015). Zhao et al. (2005) proposed that the highly creative people can maintain a positive attitude and confidence in entrepreneurship. At the same time, creativity is an essential factor that affects entrepreneurial intentions (Tantawy et al., 2021). Hu et al. (2018) pointed out that creativity is a significant predictor variable in entrepreneurship, and creativity is beneficial to individual entrepreneurship. Feldman and Bolino (2000) found that creative people have high entrepreneurial intentions. Moreover, some researchers have proposed through empirical research that creativity has a positive and significant impact on entrepreneurial intentions (Smith et al., 2016; Bellò et al., 2018; Shi et al., 2020; Murad et al., 2021). Therefore, this research proposes the following hypothesis:

H3: The creativity of college students exerts a significant positive effect on entrepreneurial intention.

### The Mediating Role of Creativity Between Personality Traits and Entrepreneurial Intention

The evidence cited in this study on the relationship between personality traits, creativity, and entrepreneurial intentions indicates that creativity may be one of the mediating variables between personality traits and entrepreneurial intentions. However, this factor has not received enough attention in the past. In previous studies, creativity was used as a mediating variable to explore the mediating role of creativity between the knowledge process and corporate performance (Imran et al., 2018). Creativity plays a mediating role between reform, openness, and the self-efficacy of innovative culture (Danish et al., 2019). Abdulaal and Nordin (2020) found that creativity has a mediating role between knowledge and human resource management. Creativity also plays a significant mediating role between training and organizational innovation (Chaubey et al., 2021). In addition, creativity plays a mediating role between participatory management and teacher job satisfaction (Zavvar et al., 2021). In summary, creativity has played a critical mediating role in past empirical research. Moreover, Fatoki (2010) pointed out that creativity is the key driving force for entrepreneurial intentions and an essential process for the formation of entrepreneurship. Based on the arguments of Hypotheses 1 and 3 and the aforementioned discussion, the present research provides a preliminary understanding of the relationship between personality traits, creativity, and entrepreneurial intention. This research aims to verify that the relationship between personality traits and entrepreneurial intentions may affect the mediating role of creativity. Therefore, this research puts forward the following hypothesis:

H4: The creativity of college students plays a mediating role between personality traits and entrepreneurial intention.

## Materials and Methods

### Research Framework

**FIGURE 1:**
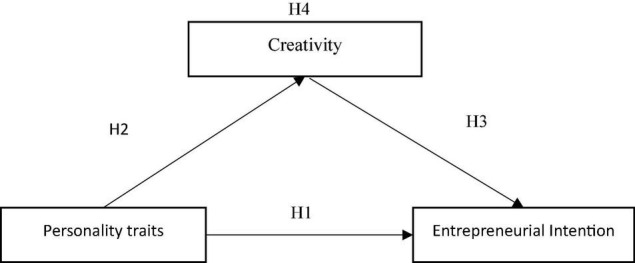
Research framework.

In this study, college students’ personality traits, entrepreneurial intention, and creativity were used as independent variable, dependent variable, and mediating variable, respectively. Based on the research hypotheses, we propose a research framework (Figure 1).

### Data Collection and Sample

In this study, we conducted a questionnaire survey on the personality traits, creativity, and entrepreneurial intention of college students in two universities in Kunming, Yunnan Province. Notably, it should be noted that both the universities are the typical experience demonstration universities of innovation and entrepreneurship in China (The State Council the People’s Republic of China, 2019).

### Pilot Test Sample Adopted in the Study

A preliminary questionnaire was developed based on the existing validated scale, and convenience sampling was adopted in two universities in Yunnan Province, China, from 1 September, 2020 to 10 September, 2020. The personality traits, creativity, and entrepreneurial intention were determined through the questionnaire survey. According to the principle of 3–5 times of the most subscale questions in the questionnaire (DeVellis, 2016), because the most topic in this study is the personality trait quality scale, a total of 40 questions, therefore, 200 volunteers were selected for a pilot study, which was in line with the sampling The standard of the number of people. The pilot-test analysis revealed the following results: In terms of the Big Five Personality Scale, the exploratory factor analysis results showed that after excluding questions with factor loading less than 0.4, a total of 35 questions after deleting the questions, factor loading between 0.410–0.805, in line with Guadagnoli and Velicer (1988) proposed that the factor loading > 0.4 can be regarded as a stable standard, the cumulative percentage of variance was 67.951%, and the validity was good; the reliability analysis results showed the following Cronbach’s α of neurotic = 0.918, Cronbach’s α of conscientiousness = 0.907, Cronbach’s α of agreeableness = 0.866, Cronbach’s α of open = 0.931, Cronbach’s α of extraversion = 0.920, with good reliability. In terms of creating a power scale, the results of exploratory factor analysis showed that: excluding the questions with factor loading less than 0.4, a total of 14 questions were deleted, factor loading = 0.418–0.792, in line with the standard, the cumulative percentage of variance was 68.346%, and the validity was good. Reliability analysis results showed: Cronbach’s α of divergent thinking = 0.842, Cronbach’s α of intelligence application ability = 0.863, Cronbach’s α of personality trait = 0.726, good reliability. In terms of the entrepreneurial intention vector table, the exploratory factor analysis results showed: factor loading = 0.488–0.770, which met the standard, the cumulative percentage of variance was 61.797%, and the validity was good. The reliability analysis result showed that Cronbach’s α of entrepreneurial intention = 0.928, with good reliability.

### Sampling and Subjects

The formal questionnaire in this study was completed between 20 October, 2020 and 30 October, 2020. Teachers assisted in the study by providing electronic questionnaires to the participants in the class. Additionally, necessary information such as research purpose, voluntariness, and anonymity was provided to the participants.

A total of 700 questionnaires were distributed. According to the proportion, 50% of the college students from each of the two universities were selected as the interviewees. After excluding invalid questionnaires, the number of final valid questionnaires was 674, and the effective response rate was 96%. According to a study in Israel (1992), the sample size was calculated using the following formula: Sample size = z2 × *p*[1 − p]/e2/1 + [z2 × *p*(1 − p)/e2N], *z* = 1.65, *N* = 62,000, *p* = 0.5, e2 = 0.0025. The sample size of the study is approximately 382, which met the standard sample size. Of the total participants, 357 (53%) participants were men and 317 (47%) participants were women. Additionally, 95 (14.1%) participants were in the freshman year, 145 (21.5%) participants were in the sophomore year, and 168 (24.9%) participants were in the junior year. There were 266 (39.5%) seniors; 324 (48.1%) participants were from liberal arts background and 350 (51.9%) participants were from science background.

### Measurement

The questionnaire comprised multiple factors containing personality traits scale, creativity scale, and entrepreneurial intention scale.

### Big-Five Personality Scale

The Big Five Personality Simplified Scale (John et al., 1991) was used in this study and revised from Wang et al. (2011) to the Chinese Big Five Personality Inventory brief version, suitable for Chinese college students. The scale comprised 40 questions, which involved five dimensions, namely neuroticism, conscientiousness, agreeableness, openness, and extroversion. Likert’s six-point scale was used for scoring, and reverse questions were used for reverse scoring.

After deleting the questions, the formal scale comprised a total of 35 questions. The collected data were subjected to confirmatory factor analysis. The results showed the following: RMR = 0.056, GFI = 0.815, NFI = 0.874, IFI = 0.900, CFI = 0.899, RMSEA = 0.083, PNFI = 0.808, and PGFI = 0.711, indicating that the fitness of the measurement model was acceptable (McDonald and Ho, 2002). Furthermore, CR of neuroticism = 0.932, CR of conscientiousness = 0.909, CR of agreeableness = 0.869, CR of openness = 0.930, and CR of extroversion = 0.920, representing the values greater than the reference CR of 0.6 (Fornell and Larcker, 1981). In addition, AVE of neuroticism = 0.660, AVE of conscientiousness = 0.589, AVE of agreeableness = 0.572, AVE of openness = 0.628, and AVE of extroversion = 0.593, which represented the values greater than the reference AVE of 0.5 (Anderson and Gerbing, 1988), indicating good convergence validity. Reliability analysis results showed that the Cronbach’s α of neuroticism = 0.931, Cronbach’s α of conscientiousness = 0.907, Cronbach’s α of agreeableness = 0.866, Cronbach’s α of openness = 0.937, and Cronbach’s α of extroversion = 0.906, which indicated good reliability.

### Creativity Scale

This study used the creativity scale (He et al., 2015), which comprises 16 questions and the following three dimensions: divergent thinking, intellectual application ability, and personality characteristics. The scale uses a five-point scoring method.

After deleting the questions, the formal scale had 14 questions. The collected data were subjected to confirmatory factor analysis. According to the results, RMR = 0.035, GFI = 0.917, NFI = 0.930, IFI = 0.942, CFI = 0.942, RMSEA = 0.081, PNFI = 0.756, PGFI = 0.646, indicating that the fitness of the measurement model is acceptable. Moreover, CR of divergent thinking = 0.851, CR of intellectual application ability = 0.862, CR of personality characteristics = 0.850, AVE of creative thinking = 0.493, AVE of intelligence application ability = 0.568, and AVE of personality traits = 0.653, thereby conforming to the Fornell and Larcker (1981) recommendation that AVE > 0.36 is the barely acceptable standard. Cronbach’s α of divergent thinking = 0.828, Cronbach’s α of intellectual application ability = 0.872, Cronbach’s α of personality characteristics = 0.843, in all dimensions of the creative power scale demonstrated good reliability.

### Entrepreneurial Intention Scale

This study used the individual entrepreneurial intention scale (Thompson, 2009), which comprises 10 questions. The scale utilizes a five-point scoring method, where reverse scoring is applied for reverse questions.

The formal scale comprised 10 questions. Confirmatory factors were used to analyze the collected data. According to the results, RMR = 0.049, GFI = 0.931, NFI = 0.947, IFI = 0.955, CFI = 0.954, RMSEA = 0.094, PNFI = 0.737, PGFI = 0.742, which indicated that the fitness of the measurement model was acceptable. The CR of entrepreneurial intention = 0.930 and AVE of entrepreneurial intention = 0.578, and the convergence validity was good. The entrepreneurial intention vector table Cronbach’s α = 0.928, which indicated good reliability.

### Data Analysis

SPSS software was used to analyses the common method deviation of personality traits, creativity, and entrepreneurial intentions. Then, by using the correlation analysis, the relationship among these three main variables was determined. Finally, the direct influence of personality traits and creativity on entrepreneurial intentions was determined, and the mediating effect of creativity under the influence of personality traits on entrepreneurial intentions was analyzed.

### Common Method Deviation Test

To test common method bias, single-factor testing is used (Harman, 1976). The characteristic roots of 9 factors in the present study are greater than 1. the first factor can only explain 11.324%, which is far less than the critical value of 40%. Therefore, no serious common method bias problem exists in this study.

## Results

### Descriptive Statistics and Correlation Analysis

Descriptive statistics suggest that college students’ personality traits, creativity, and entrepreneurial intention are all at the upper-middle level. A Pearson correlation analysis indicates the existence of a significant correlation among the variables. The correlation analysis variables between openness and extraversion, openness and creativity, and conscientiousness and entrepreneurial intention are relatively high and consequently require further discriminative validity analysis.

The results of this study demonstrate that the AVE value of the two variables is greater than the square value of the correlation coefficient between the two variables. This satisfies the criteria for evaluating the validity of the difference (Fornell and Larcker, 1981). As shown in Table 1, any two variables are characterized by good discriminative validity.

**TABLE 1:** Descriptive analysis and correlation analysis.

	M	SD	1	2	3	4	5	6	7
1	2.081	1.089	**0.812**						
2	5.089	0.869	−0.718***	**0.767**					
3	5.046	0.865	−0.595***	0.716***	**0.756**				
4	5.001	4.985	−0.676***	0.782***	0.746***	**0.792**			
5	4.985	0.988	−0.696***	0.748***	0.712***	0.832***	**0.770**		
6	4.287	0.643	−0.649***	0.742***	0.669***	0.810***	0.757***	**0.808**	
7	4.945	0.996	−0.719***	0.814***	0.694***	0.779***	0.789***	0.790***	**0.760**

***p < 0.001; Bolded fonts are AVE root values; 1 = Neuroticism, 2 = Conscientiousness, 3 = agreeableness, 4 = Openness, 5 = Extraversion, 6 = Creativity, 7 = Entrepreneurial Intention.

### Regression Analysis

Multiple regression analyses serve to verify the hypothesis. Variance inflation factor (VIF) is used to test for multicollinearity. The results of previous empirical studies suggest that differences in gender, majors, and grades can cause significant differences in personality traits (Russo and Stol, 2020), including creativity (Vedel et al., 2015; Zia and Rouhollahi, 2020; He and Wong, 2021; Said-Metwaly et al., 2021) and entrepreneurial intention (Abba et al., 2021; Dao et al., 2021; Gurel et al., 2021). The *t* test and ANOVA test yield the following results. There is initially a significant difference between gender in entrepreneurial intention, where boys have higher results than girls. Second, there is a significant difference in entrepreneurial intention in majors where science students score higher than liberal arts. Third, significant differences exist in entrepreneurial intention in grades (*F* = 49.789, *p*<0.001), among which a freshman is higher than a sophomore, a junior is higher than a sophomore, a junior is higher than a senior, and a senior is higher than a sophomore. Gender, major, and grade are therefore chosen as control variables in this study.

The mediating role of creativity in college students between personality traits and entrepreneurial intention was tested by controlling the influence of gender, major, and grade. As shown in Table 2, in Model 1, neuroticism has a significant negative effect on entrepreneurial intention (β = –0.143, *p* < 0.001). Conversely, conscientiousness exerts a significant positive effect on entrepreneurial intention (β = 0.367, *p* < 0.001). agreeableness on the other hand has no significant effect on entrepreneurial intention (β = 0.054, *p* > 0.05). Openness generates a significant positive effect on entrepreneurial intention (β = 0.117, *p* < 0.01). Extraversion has a significant positive effect on entrepreneurial intention (β = 0.229, *p* < 0.001). H1 is consequently established.

**TABLE 2:** Examination of the mediating role of creativity in the influence of personality traits on entrepreneurial intention.

Variable	Model 1	Model 2	Model 3
	
	EI	Creativity	EI
	
	β	β	β
Gender (Boys)	−0.067**	0.001	−0.067**
Major (liberal arts)	0.029	0.026	0.022
Grade 1 (Freshman)	0.007	0.114***	–0.021
Grade 2 (Sophomore)	−0.066**	–0.038	−0.057**
Grade 3 (Junior)	0.037	0.150***	0.000
Neuroticism	−0.143***	−0.066*	−0.126***
Conscientiousness	0.367***	0.198***	0.318***
Agreeableness	0.054	0.036	0.045
Openness	0.117**	0.417***	0.014
Extraversion	0.229***	0.141**	0.194***
**Creativity**			0.247***
*R* ^2^	0.764	0.730	0.780
Adj *R*^2^	0.760	0.725	0.776
*F*	214.137***	178.853***	213.425***

*p < 0.05, **p < 0.01, ***p < 0.001; Gender, major, and grade are dummy variables.

*Boys are the experimental group within the gender group, while the girls are the reference group.*

*Liberal arts are the experimental group, while sciences are the reference group.*

*Freshmen, sophomores, and juniors are the experimental group in the grade group, while seniors are the reference group; EI, Entrepreneurial Intention.*

As presented in Model 2, neuroticism has a significant negative effect on creativity (β = –0.066, *p* < 0.05). Conscientiousness has a significant positive effect on creativity (β = 0.198, *p* < 0.001). Further, agreeableness has no significant effect on creativity (β = 0.036, *p* > 0.05). Openness exerts a significant positive effect on creativity (β = 0.417, *p* < 0.001). Extraversion shows a significant positive effect on creativity (β = 0.141, *p* < 0.001). H2 is established as a result.

The mediator creativity is added in Model 3. Creativity has a significant positive effect on entrepreneurial intention (β = 0.247, *p* < 0.001), thus establishing H3. When the β value of neuroticism on entrepreneurial intention decreases from –0.143 to –0.126, it reaches a significant level (β = –0.126, *p* < 0.001). Therefore, creativity plays a partial mediating role between neuroticism and entrepreneurial intention. The β value of conscientiousness on entrepreneurial intention reaches a significant level when it decreases from 0.367 to 0.318 (β = 0.318, *p* < 0.001). Therefore, creativity plays a partial mediating role between conscientiousness and entrepreneurial intention, and the significant effect of openness on entrepreneurial intention disappears (β = 0.014, *p* > 0.05). Creativity plays a complete mediating role between openness and entrepreneurial intention. The β value of extraversion on entrepreneurial intention reaches a significant level when it decreases from 0.229 to 0.194 (β = 0.194, *p* < 0.001). Therefore, creativity plays a partial mediating role between extraversion and entrepreneurial intention. H4 is thus established.

In this study, The Sobel test is used in this study to further test the mediation effect (Sobel, 1982), which calculates the unstandardized regression coefficients and standard errors. A value of Z greater than 1.96 represents a significant mediation effect. The results show that *Z* = 23.317, *p* < 0.001, indicating that creativity plays a mediating role between personality traits and entrepreneurial intention. In Model 3, VIF is between 1.254–5.128, which is lower than the standard value of 10. This result suggests a lack of a serious collinearity problem (HairJr., Anderson et al., 1995), as shown in Table 2.

## Discussion and Conclusion

### Theoretical Contributions

First, the results of this study indicated that college students’ neuroticism has a significant negative impact on their entrepreneurial intentions. Besides, conscientiousness, openness, and extraversion have a significant positive impact on entrepreneurial intention, which is similar to the results obtained by Western empirical studies (Murugesan and Jayavelu, 2017; Israr and Saleem, 2018; Şahin et al., 2019; Kristanto and Pratama, 2020; Liu D. et al., 2021). The following conclusions can therefore be inferred. College students with low neuroticism have relatively fewer negative emotions such as anxiety and depression and are more able to withstand the obstacles brought by entrepreneurship. Furthermore, those with high conscientiousness have high self-demand, self-control, execution along a cautious attitude toward entrepreneurship. Students with high openness show strong curiosity, imagination, and cooperation and have innovative and open thinking about entrepreneurship. They are also talented at accepting suggestions from others. Students with high extroversion are passionate and fond of social activities, which allows them to make a wide range of entrepreneurial connections. Agreeableness has no significant effect on entrepreneurial intention, which is different from the conclusions of Western studies. Due to the cultural differences between China and the West, Chinese college students with high agreeableness show characteristics such as compassion and willingness to help others, which are not conducive to entrepreneurship.

Furthermore, the results of this study indicated that college students’ neuroticism has a significant negative impact on their creativity. Additionally, conscientiousness, openness, and extraversion have a significant positive impact on creativity. This is in line with the results of Western empirical studies (Jirásek and Sudzina, 2020; Sadana et al., 2021; Sarma and Borooah, 2021). This study concludes that college students with low neuroticism are characterized by compulsive dependence and are more capable of using their creativity (Albert and Runco, 1987). In other words, college students with low neuroticism do not rely on help from others. Active thinking is conducive to divergent thinking and increases creativity. College students with high conscientiousness pay attention to details, do their work thoroughly and pursue achievement with creativity (McCrae, 1987). Agreeableness has nothing to do with creativity (Silvia et al., 2011). Furthermore, the higher the agreeableness, the lower the creativity (King et al., 1996). Conversely, negative qualities such as arrogance and hostility accompany high creativity (Feist, 1999). Being open-minded and curious makes it easier to gain new ideas (Goldberg, 1990). People with openness are characterized by flexible thinking and are willing to accept new ideas (Zhao and Seibert, 2006). Moreover, they are more capable of getting rid of traditional customs and adopting new and unique ways of thinking (Sung and Choi, 2009). People with extraversion are usually enthusiastic and sociable, which contributes to inspiring creativity (Prabhu et al., 2008).

Second, the results of this study indicated that the creativity of college students has a significant positive effect on entrepreneurial intentions, which is consistent with the results of Western empirical research (Hu et al., 2018; Entrialgo and Iglesias, 2020). This study concludes that college students with high creativity have novel ideas and innovative abilities. Innovative thinking can effectively develop available resources, while creative ideas make a positive contribution to entrepreneurial intention (Biraglia and Kadile, 2017; Murad et al., 2021).

Finally, the study results of the study also suggest that creativity plays a partial mediating role between neuroticism, conscientiousness, extraversion, and entrepreneurial intention, while creativity plays a complete mediating role between openness and entrepreneurial intention. This result is consistent with the conclusions of previous empirical studies (Wang et al., 2018). College students with high creativity thus have divergent thinking and innovative thinking, consequently having more ideas to start new companies (Anjum et al., 2021).

### Practical Implications

The research results further provide a reference value for the improvement and optimization of entrepreneurial practice. First, colleges and universities should perform relevant tests to understand personality traits when college students enroll. For college students with neuroticism, teachers can relieve their psychological pressure through psychological counseling. College students with conscientiousness should be encouraged to give lectures in class to strengthen their divergent logical thinking skills. In terms of college student’s openness, teachers can motivate them to participate in the planning of class activities and the design of classroom training. In addition, activities such as scenario simulation and role-playing can train openness thinking and strengthen creativity. For college students with extraversion, they should be encouraged to participate in social activities, class organizations, or clubs. Through the above-mentioned teaching of personality traits in accordance with their aptitude, the entrepreneurship and creativity of college students can be significantly enhanced. Secondly, Colleges and universities should therefore create a diversified and open learning environment, conduct lectures that cultivate creativity and promote reading education. Furthermore, teachers should encourage students’ enthusiasm by designing interesting educational interactions, such as group discussions, and affirm their uniqueness by encouraging students. At last, teachers should focus on students’ different neuroticism, conscientiousness, extraversion, and openness to promote entrepreneurial intention and cultivate creativity.

### Limitations and Suggestions for Future Research

It is necessary to expand the sample range for future research. The relationship among personality traits, creativity, and entrepreneurial intention of college students can be deeply explored through qualitative in-depth interviews or a combination of quantitative and qualitative studies. Past research results show that goal orientation theory has been applied to creativity-related research (Huang and Luthans, 2015; Lee and Yang, 2015; Kaspi-Baruch, 2019). Therefore, follow-up research can apply goal orientation theory as well to explore the relationship among personality traits, creativity, and entrepreneurial intention. This study adopts the Big-Five Personality Traits Scale. A subjective personality has a positive and significant effect on entrepreneurial intention (Hu et al., 2018; Kumar and Shukla, 2019; Li et al., 2020), while a narcissistic personality has a positive and significant effect on entrepreneurship (Al-Ghazali and Afsar, 2021; Liu H. C. et al., 2021). Future research can consider other specific personality trait scales to provide more empirical evidence for related research in the field of entrepreneurship.

## Data Availability Statement

The raw data supporting the conclusion of this article will be made available by the authors, without undue reservation.

## Ethics Statement

The studies involving human participants were reviewed and approved by Dhurakij Pundit University. The patients/participants provided their written informed consent to participate in this study. Written informed consent was obtained from the individual(s) for the publication of any potentially identifiable images or data included in this article.

## Author Contributions

L-NL conceived the study idea, edited the data, performed the analysis and interpretation, drafted the skeleton of the manuscript, and critically reviewed the manuscript. J-HH contributed to constructing the model, interpreting the model results, and intensively editing the language of the manuscript. S-YG participated in the revision of the manuscript. All authors read and approved the final manuscript and participated in the critical appraisal as well as revision of the manuscript.

## Conflict of Interest

The authors declare that the research was conducted in the absence of any commercial or financial relationships that could be construed as a potential conflict of interest.

## Publisher’s Note

All claims expressed in this article are solely those of the authors and do not necessarily represent those of their affiliated organizations, or those of the publisher, the editors and the reviewers. Any product that may be evaluated in this article, or claim that may be made by its manufacturer, is not guaranteed or endorsed by the publisher.
